# Supplementary material for: Mps2 links Csm4 and Mps3 to form a telomere-associated LINC complex in budding yeast
Source: Life Sci Alliance. 2020 Sep 23;3(12):e202000824. doi: 10.26508/lsa.202000824 (PMC7536833; doi:10.26508/lsa.202000824)
Supplement: Supplementary file 1 [file LSA-2020-00824_TableS1.docx]

**Table S1. Yeast strains used in this study**

| Strain/background | | Genotype | Experiment |
| --- | --- | --- | --- |
| HY5691 | SK1 | *his3Δ200, leu2-k, ura3, lys2, ho::LYS2, TAP-CSM4, ndt80∆::HB, GFP-MPS3::LEU2//his3Δ200, leu2-k, ura3, lys2, ho::LYS2, TAP-CSM4, ndt80∆::HB, GFP-MPS3::LEU2* | Fig. 1B |
| HY5971 | SK1 | *leu2, his3, ura3, ndt80∆::KAN, TAP-MPS2, V5-CSM4//leu2, his3, ura3, ndt80∆::KAN, TAP-MPS2, V5-CSM4* | Fig. 1C |
| HY5970 | SK1 | *his3Δ200, leu2-k, ura3, lys2, ho::LYS2, TAP-CSM4, ndt80Δ::HB, GFP-MPS3::LEU2, MPS2-3HA::HIS5//his3Δ200, leu2-k, ura3, lys2, ho::LYS2, TAP-CSM4, ndt80Δ::HB, GFP-MPS3::LEU2, MPS2-3HA::HIS5* | Fig. 1C |
| HY6222 | SK1 | *his3Δ200, leu2-k, ura3, lys2, ho::LYS2, P_DMC1_-Apple-MPS2::LEU2, GFP-CSM4//his3Δ200, leu2-k, ura3, lys2, ho::LYS2, P_DMC1_-mApple-MPS2, GFP-Csm4* | Fig. 1E |
| HY6183 | SK1 | *his3Δ200, leu2-k, ura3, lys2, ho::LYS2, MPS3-V5::HIS5, ndt80∆::KAN, TAP-MPS2//his3Δ200, leu2-k, ura3, lys2, ho::LYS2, MPS3-V5::HIS5, ndt80∆::KAN, TAP-MPS2* | Fig. 1D |
| HY6184 | SK1 | *his3Δ200, leu2-k, ura3, lys2, ho::LYS2, V5-MPS2::HIS5, ndt80∆::KAN, TAP-MPS3//his3Δ200, leu2-k, ura3, lys2, ho::LYS2, V5-MPS2::HIS5, ndt80∆::KAN, TAP-MPS3* | Fig. 1D |
| HY5825 | SK1 | *his3Δ200, leu2-k, ura3, lys2, ho::LYS2, GFP-MPS2, TUB4-mAPPLE::HIS5//his3Δ200, leu2-k, ura3, lys2, ho::LYS2, GFP-MPS2, TUB4-mAPPLE::HIS5* | Fig. 2A |
| HY5764 | SK1 | *his3Δ200, leu2-k, ura3, lys2, ho::LYS2, MPS2-3HA::HIS5//his3Δ200, leu2-k, ura3, lys2, ho::LYS2, MPS2-3HA::HIS5* | Fig. 2B |
| HY5743 | SK1 | *his3Δ200, leu2-k, ura3, lys2, ho::LYS2, P_CLB2_-MPS2::HB//his3Δ200, leu2-k, ura3, lys2, ho::LYS2, P_CLB_2-MPS2::HB* | Fig. 2B |
| HY1635 | SK1 | *his3Δ200, leu2-k, ura3, lys2, ho::LYS2, TUB4-mAPPLE::HIS5//his3Δ200, leu2-k, ura3, lys2, ho::LYS2, TUB4-mAPPLE::HIS5* | Figs. 2C, S1A, S1B |
| HY5775 | SK1 | *his3Δ200, leu2-k, ura3, lys2, ho::LYS2, TUB4-mApple::HIS5, P_CLB2_-MPS2::HB//his3Δ200, leu2-k, ura3, lys2, ho::LYS2, TUB4-mApple::HIS5, P_CLB2_-MPS2::HB* | Figs. 2C, S1D |
| HY5948 | SK1 | *his3∆, leu2, lys2, ho::LYS2, ura3, MPS3-mApple::HIS5, GFP-MPS2//his3∆, leu2, lys2, ho::LYS2, ura3, MPS3-mApple::HIS5, GFP-MPS2* | Fig. 2D |
| HY5965 | SK1 | *his3Δ200, leu2-k, ura3, lys2, ho::LYS2, GFP-MPS2, csm4∆::HB, MPS3-mApple::HIS5//his3Δ200, leu2-k, ura3, lys2, ho::LYS2, GFP-MPS2, csm4∆::HB, MPS3-mApple::HIS5* | Fig. 2D |
| HY5966C | SK1 | *his3Δ200, leu2-k, ura3, lys2, ho::LYS2, GFP-CSM4, MPS3-mApple::HIS5//his3Δ200, leu2-k, ura3, lys2, ho::LYS2, GFP-CSM4, MPS3-mApple::HIS5* | Fig. 2E |
| HY5966-2 | SK1 | *his3Δ200, leu2-k, ura3, lys2, ho::LYS2, GFP-CSM4, MPS3-mApple::HIS5, P_CLB2_-MPS2::HB//his3Δ200, leu2-k, ura3, lys2, ho::LYS2, GFP-CSM4, MPS3-mApple::HIS5, P_CLB2_-MPS2::HB* | Fig. 2E |
| HY5895 | SK1 | *leu2, ura3, REC8-GFP::HIS5, MPS2-3HA::HIS5, NDJ1-V5::HIS5//leu2, ura3, REC8-GFP::HIS5, MPS2-3HA::HIS5, NDJ1-V5::HIS5* | Fig. 3A |
| HY5959 | SK1 | *leu2, ura3, REC8-GFP::HIS5, MPS2-3HA::HIS5, V5-CSM4//leu2, ura3, REC8-GFP::HIS5, MPS2-3HA::HIS5, V5-CSM4* | Fig. 3B |
| HY5794 | SK1 | *leu2, ura3, REC8-GFP::HIS5, MPS2-3HA::HIS5//leu2, ura3, REC8-GFP::HIS5, MPS2-3HA::HIS5* | Fig. 3C |
| HY5867-2 | SK1 | *leu2, ura3, REC8-GFP::HIS5, MPS2-3HA::HIS5, csm4Δ::HB//leu2, ura3, REC8-GFP::HIS5, MPS2-3HA::HIS5, csm4Δ::HB* | Fig. 3C |
| HY5849 | SK1 | *leu2, ura3, REC8-GFP::HIS5, MPS2-3HA::HIS5, P_CLB2_-MPS3::NAT//leu2, ura3, REC8-GFP::HIS5, MPS2-3HA::HIS5, P_CLB2_-MPS3::NAT* | Fig. 3C |
| HY5845 | SK1 | *leu2, ura3, REC8-GFP::HIS5, MPS2-3HA::HIS5, ndj1Δ::HB//leu2, ura3, REC8-GFP::HIS5, MPS2-3HA::HIS5, ndj1Δ::HB* | Fig. 3C |
| HY5917 | SK1 | *his3Δ200, leu2-k, ura3, lys2, ho::LYS2, V5-CSM4, REC8-GFP::HIS5//his3Δ200, leu2-k, ura3, lys2, ho::LYS2, V5-CSM4, REC8-GFP::HIS5* | Fig. 3D |
| HY5982 | SK1 | *ho::hisG, leu2::hisG, lys2, ura3, arg4-bgl, P_CLB2_-MPS2::HB, REC8-GFP::HIS5, V5-CSM4//ho::hisG, leu2::hisG, lys2, ura3, arg4-bgl, P_CLB2_-MPS2::HB, REC8-GFP::HIS5, V5-CSM4* | Fig. 3D |
| HY4494 | SK1 | *ura3, leu2, RAP1-GFP::HIS5, TUB4-mAPPLE::HIS5//ura3, leu2, RAP1-GFP::HIS5, TUB4-mAPPLE::HIS5* | Figs. 4A, 4B |
| HY5795 | SK1 | *his3Δ200, leu2-k, ura3, lys2, ho::LYS2, P_CLB2_-MPS2::HB, RAP1-GFP::HIS5, TUB4-mAPPLE::HIS5//his3Δ200, leu2-k, ura3, lys2, ho::LYS2, P_CLB2_-MPS2::HB, RAP1-GFP::HIS5, TUB4-mAPPLE::HIS5* | Figs. 4A, 4B |
| HY6173 | SK1 | *arg4-Nsp, his4-x, leu2, trp1, ura3, csm4Δ::NAT, RAP1-GFP::HIS5, TUB4-mAPPLE::HIS5//arg4-Nsp, his4-x, leu2, trp1, ura3, csm4Δ::NAT, RAP1-GFP::HIS5, TUB4-mAPPLE::HIS5* | Figs. 4A, 4B |
| HY6170 | SK1 | *arg4-Nsp, his4-x, leu2, trp1, ura3, csm4Δ::NAT, P_CLB2_-MPS2::HB, RAP1-GFP::HIS5, TUB4-mAPPLE::HIS5//arg4-Nsp, his4-x, leu2, trp1, ura3, csm4Δ::NAT, P_CLB2_-MPS2::HB, RAP1-GFP::HIS5, TUB4-mAPPLE::HIS5* | Figs. 4A, 4B |
| HY1298C | SK1 | *arg4-Nsp, his4-x, leu2, trp1, ura3//arg4-Bgl, his4-B, leu2, trp1, ura3* | Figs. 4C-E |
| HY5776 | SK1 | *arg4-Nsp, his4-x, leu2, trp1, ura3, P_CLB2_-MPS2::HB//arg4-Bgl, his4-B, leu2, trp1, ura3, P_CLB2_-MPS2::HB* | Figs. 4C-E |
| HY5791 | SK1 | *arg4-Nsp, his4-x, leu2, trp1, ura3, csm4Δ::NAT//arg4-Bgl, his4-B, leu2, trp1, ura3, csm4Δ::NAT* | Figs. 4C-E |
| BY4741 | S288C | *MATa, his3∆1, leu2∆0, met15∆0, ura3∆0* | Fig. 5A |
| HY4242 | S288C | *MATa, his3∆1, leu2∆0, met15∆0, ura3∆0, CSM4::P_GAL1_-V5-CSM4::URA3* | Figs. 5A, S4A |
| HY5599-A | S288C | *MATa, his3∆1, leu2∆0, met15∆0, ura3∆0, MPS2::P_GAL1_-GFP-MPS2::LEU2* | Fig. 5A |
| HY5694-2A | S288C | *MATa, his3∆1, leu2∆0, met15∆0, ura3∆0, CSM4::P_GAL1_-V5-CSM4::URA3, MPS2:: P_GAL1_-GFP-MPS2::LEU2* | Fig. 5A |
| HY4985 | S288C | *MATa, his3∆1, leu2∆0, lys2∆0, ura3∆0, TUB4-mApple::HIS5, MPS3-GFP::HIS5, CSM4::P_GAL1_V5-CSM4::URA3* | Figs. 5C, 6A |
| HY6559 | S288C | *MATa, his3∆1, leu2∆0, met15∆0, ura3∆0, TUB4-mApple::HIS5, RAP1-GFP::HIS5, CSM4::P_GAL1_-V5-CSM4::LEU2, NDJ1::P_GAL1_-NDJ1::URA3* | Fig. 6B |
| HY6093 | SK1 | *arg4, ura3, leu2, spo11-Y135F::HB, TUB4-GFP::HIS5//arg4, ura3, leu2, spo11-Y135F::HB, TUB4-GFP::HIS5* | Fig. S1C |
| HY5999 | SK1 | *arg4, ura3, leu2, spo11-Y135F::HB, TUB4-GFP::HIS5, P_CLB2_-MPS2::HB, // arg4, ura3, leu2, spo11-Y135F::HB, TUB4-GFP::HIS5, P_CLB2_-MPS2::HB* | Fig. S1E |
| HY5790 | SK1 | *arg4, leu2, ura3, his4, csm4∆::HB, TUB4-mApple::HIS5//arg4, leu2, ura3, his4, csm4∆::HB, TUB4-mApple::HIS5* | Fig. S1F |
| HY6094 | SK1 | *arg4, leu2, ura3, his4, csm4∆::HB, TUB4-mApple::HIS5, spo11-Y135F::HB//arg4, leu2, ura3, his4, csm4∆::HB, TUB4-mApple::HIS5, spo11-Y135F::HB* | Fig. S1G |
| HY5825 | SK1 | *his3Δ200, leu2-k, ura3, lys2, ho::LYS2, GFP-MPS2, TUB4-mApple::HIS5//his3Δ200, leu2-k, ura3, lys2, ho::LYS2, GFP-MPS2, TUB4-mApple::HIS5* | Fig. S2A |
| HY6188 | SK1 | *leu2, ura3, TUB4-mApple::HIS5, GFP-MPS2, P_CLB2_-MPS3::NAT//leu2, ura3, TUB4-mAppleE::HIS5, GFP-MPS2, P_CLB2_-MPS3::NAT* | Fig. S2A |
| HY6435 | SK1 | *his3Δ200, leu2-k, ura3, lys2, ho::LYS2, NUP49-mApple::HIS5//his3Δ200, leu2-k, ura3, lys2, ho::LYS2, NUP49-mApple::HIS5* | Fig. S2B |
| HY6304 | SK1 | *his3Δ200, leu2-k, ura3, lys2, ho::LYS2, P_CLB2_-MPS3::NAT, NUP49-mApple::HIS5//his3Δ200, leu2-k, ura3, lys2, ho::LYS2, P_CLB2_-MPS3::NAT, NUP49-mApple::HIS5* | Fig. S2B |
| HY6545 | SK1 | *leu2, lys2, ura3, arg4-bgl, REC8-GFP::HIS5, MPS3-V5::HIS5// leu2, lys2, ura3, arg4-bgl, REC8-GFP::HIS5, MPS3-V5::HIS5* | Fig. S2C |
| HY6544 | SK1 | *leu2, lys2, ura3, arg4-bgl, P_CLB2_-MPS2::HB, REC8-GFP::HIS5, MPS3-V5::HIS5//leu2, lys2, ura3, arg4-bgl, P_CLB2_-MPS2::HB, REC8-GFP::HIS5, MPS3-V5::HIS5* | Fig. S2C |
| HY6471 | SK1 | *his3Δ200, leu2-k, ura3, lys2, ho::LYS2, LYS4::TetO::HIS5, ndt80∆::KAN, TetR-GFP::LEU2//his3Δ200, leu2-k, ura3, lys2, ho::LYS2, LYS4::TetO::HIS5, ndt80∆::KAN, TetR-GFP::LEU2* | Fig. S2D |
| HY6079 | SK1 | *his3Δ200, leu2-k, ura3, lys2, ho::LYS2, P_CLB2_-MPS2::HB, LYS4::TetO::HIS5, ndt80∆::KAN//his3Δ200, leu2-k, ura3, lys2, ho::LYS2, P_CLB2_-MPS2::HB, LYS4::TetO::HIS5, ndt80∆::KAN, TETR-GFP::LEU2* | Fig. S2D |
| HY6468 | SK1 | *his3Δ200, leu2-k, ura3, lys2, ho::LYS2, csm4Δ::NAT, LYS4::TetO::HIS5, ndt80∆::KAN//his3Δ200, leu2-k, ura3, lys2, ho::LYS2, csm4Δ::NAT, LYS4::TetO::HIS5, ndt80∆::KAN, TetR-GFP::LEU2* | Fig. S2D |
| HY6408 | S288C | *MATa, his3∆1, leu2∆0, lys2∆0, ura3∆0, NUP49-GFP::HIS5, HTA1-mApple::HIS5, CSM4::P_GAL1_-V5-CSM4::LEU2* | Fig. S3A |
| HY6191 | S288C | *MATa, his3∆1, leu2∆0, met15∆0, ura3∆0, MPS3-mApple::HIS5, GFP-MPS2, CSM4::P_GAL1_-V5-CSM4::LEU2* | Fig. S3B |
| HY6268-3C | S288C | *MATa, his3∆1, leu2∆0, lys2∆0, ura3∆0, mps2∆::HB, pom152∆::KAN* | Fig. S4A |
| HY6441 | S288C | *MATa, his3∆1, leu2∆0, lys2∆0, ura3∆0, mps2∆::HB, MPS3-GFP::HIS5, pom152∆::KAN, TUB4-MAPPLE::HIS5, CSM4::P_GAL1_-V5-CSM4::LEU2* | Figs. S4A, S4B |
