# Supplementary material for: Mps2 links Csm4 and Mps3 to form a telomere-associated LINC complex in budding yeast
Source: Life Sci Alliance. 2020 Sep 23;3(12):e202000824. doi: 10.26508/lsa.202000824 (PMC7536833; doi:10.26508/lsa.202000824)
Supplement: Supplementary file 2 [file LSA-2020-00824_TableS2.docx]

**Table S2. Plasmids used in this study**

| Plasmid name | Gene construct | Selective marker |
| --- | --- | --- |
| pHG521 | *P_CSM4_-TAP-CSM4* | *URA3* |
| pHG563 | *P_MPS2_-TAP-MPS2* | *URA3* |
| pHG564 | *P_CSM4_-V5-CSM4* | *URA3* |
| pHG581 | *P_MPS2_-V5-MPS2* | *URA3* |
| pHG562 | *P_MPS2_-GFP-MPS2* | *URA3* |
| pHG476 | *P_CSM4_-GFP-CSM4* | *LEU2* |
| pK3524 | *TetR-GFP* | *LEU2* |
| pHG334 | *P_GAL1_-V5-CSM4* | *URA3* |
| pHG527 | *P_GAL1_-GFP-MPS2* | *LEU2* |
| pHG315 | *P_GAL1_-V5-NDJ1* | *LEU2* |
| pHG317 | *P_GAL1_-V5-CSM4* | *LEU2* |
