# Supplementary material for: Mps2 links Csm4 and Mps3 to form a telomere-associated LINC complex in budding yeast
Source: Life Sci Alliance. 2020 Sep 23;3(12):e202000824. doi: 10.26508/lsa.202000824 (PMC7536833; doi:10.26508/lsa.202000824)
Supplement: Supplementary file 3 [file LSA-2020-00824_TableS3.docx]

**Table S3. Primers used in this study**

| Primer Name | Sequence |
| --- | --- |
| MPS2-tagF1 | TACCGTTCAAATGCCAATGTGGATGACGCATATAGTAGAGTATTCGGCATTGCGGCCGCTCTAGAACTAGT |
| MPS2-tagR1 | CGTGACTATTTTCACTGTGTATGAAGTGGCACGGTGCAAAGGCCAAGGTTTACCCCCTCGAGGTCGACGGTA |
| MPS2-deletionF1 | TAACGGTGCGTTTGATGCTATTTTTGAGTACGCATGGGGCCAGGGGCATGATGTGACT |
| MPS2-deletionR1 | TATGAAGTGGCACGGTGCAAAGGCCAAGGTTTAAATGCCGAAGCTCGTTTTCGACACTGGAT |
| CSM4-deletionF1 | TTCTTCCCAAAAGGCAATATTGCAGAAGAAGAACTAGAAAATCAGGGGCATGATGTGACT |
| CSM4-deletionR1 | TGCGATGACGAATTTAAGGTGTGAAGACACCTCATCGCTCGCTCGTTTTCGACACTGGAT |
| POM152-deletionF1 | ATTGGATGGGCAGTTCTGTGTCAGGATCACCACGTCCATCTTATAGCAGTCAGGGGCATGATGTGACT |
| POM152-deletionR1 | CTGTAATTTCAATCGCCTCGTAGGTACCTTGCAAGCTTGTAATTACCTTGCTCGTTTTCGACACTGGAT |
| PCLB2-MPS2F1 | TGTTCAAGAAAACGCATCATCCAAGAAAACGTGTTGTTGCTACTGGCATAGGCCACTAGTGGATCT |
| PCLB2-MPS2R1 | ATTTGGCCCCATGCGTACTCAAAAATAGCATCAAACGCACCGTTACTCATAGCAGCGTAATCTGGAACGT |
